# Infectious disease control: from health security strengthening to health systems improvement at global level

**DOI:** 10.1186/s41256-023-00319-w

**Published:** 2023-09-05

**Authors:** Xiao-Xi Zhang, Yin-Zi Jin, Yi-Han Lu, Lu-Lu Huang, Chuang-Xin Wu, Shan Lv, Zhuo Chen, Hao Xiang, Xiao-Nong Zhou

**Affiliations:** 1 School of Global Health, Chinese Center for Tropical Diseases Research, Shanghai Jiao Tong University School of Medicine, Shanghai, People’s Republic of China; 2 One Health Center, Shanghai Jiao Tong University-The University of Edinburgh, Shanghai, People’s Republic of China; 3 Department of Global Health, School of Public Health, Wuhan University, Wuhan, People’s Republic of China; 4 Global Health Institute, Wuhan University, Wuhan, People’s Republic of China; 5 Department of Global Health, School of Public Health, Peking University, Beijing, People’s Republic of China; 6 Institute for Global Health and Development, Peking University, Beijing, People’s Republic of China; 7 School of Public Health, Fudan University, Shanghai, People’s Republic of China; 8 Global Health Institute, Fudan University, Shanghai, People’s Republic of China; 9 National Institute of Parasitic Diseases at Chinese Center for Disease Control and Prevention (Chinese Center for Tropical Diseases Research), NHC Key Laboratory of Parasite and Vector Biology, WHO Collaborating Centre for Tropical Diseases, Shanghai, People’s Republic of China; 10 Department of Health Policy and Management, College of Public Health, University of Georgia, Athens, GA USA; 11 School of Economics, Faculty of Humanities and Social Sciences, University of Nottingham Ningbo China, Ningbo, Zhejiang People’s Republic of China

**Keywords:** Infectious disease control, Global health, Health security, Health system

## Abstract

Since the twenty first century, the outbreaks of global infectious diseases have caused several public health emergencies of international concern, imposing an enormous impact on population health, the economy, and social development. The COVID-19 pandemic has once again exposed deficiencies in existing global health systems, emergency management, and disease surveillance, and highlighted the importance of developing effective evaluation tools. This article outlines current challenges emerging from infectious disease control from the perspective of global health, elucidated through influenza, malaria, tuberculosis, and neglected tropical diseases. The discordance among government actors and absent data sharing platforms or tools has led to unfulfilled targets in health system resilience and a capacity gap in infectious disease response. The current situation calls for urgent action to tackle these threats of global infectious diseases with joined forces through more in-depth international cooperation and breaking governance barriers from the purview of global health. Overall, a systematic redesign should be considered to enhance the resilience of health systems, which warrants a great need to sustain capacity-building efforts in emergency preparedness and response and raises an emerging concern of data integration in the concept of One Health that aims to address shared health threats at the human-animal-environment interface.

**Supplementary Information:**

The online version contains supplementary material available at 10.1186/s41256-023-00319-w.

## Background

Global health has contributed significantly to the progress made in human health over the past century. Global health is a newly established branch of health sciences, with the task of seeking global solutions to widespread health impact issues, and an ultimate goal of improving health equity and disparities [1].

The brief history of global health can be roughly divided into the following four stages. The first, nascent stage featured a transnational quarantine system. In the 19th century, European countries implemented quarantines to protect vulnerable port cities from major epidemics such as cholera and plague [2]. The second stage was the development stage, with the main purpose of supporting international trade. To coordinate the contradiction between the transnational quarantine system and free trade, the first International Health Care Conference was held in France in 1851 and became the starting point for the establishment and institutionalization of the international health system. The third stage was the formation stage of the international health governance system, led by the World Health Organization (WHO). The establishment of the WHO marked the formation of an international health system with sovereign states as the main players. Since then, international health has rapidly developed with a focus on fostering national and international efforts to control major infectious diseases. The fourth stage consisted of comprehensive cooperation under globalization. The process of globalization made international health problems more complex and blurred national boundaries [3], thus giving birth to the concept of global health.

Global health weakens the concept of the nation as the highest administrative level and emphasizes the health development and security of all humans as diverse participants. Despite a substantial decline in the global burden of disease in the twenty first century, infectious diseases such as HIV/AIDS, malaria, tuberculosis, and diarrhea are still causing high mortality rates in developing countries due to malnutrition, crowded living conditions, poor hygiene, etc. [4]. In December 2019, the sudden outbreak of the coronavirus disease 2019 (COVID-19) quickly swept the world, further exposing the deficiencies in the existing global system for infectious disease control. For instance, the early failure of the United States COVID-19 testing kits highlighted the limits of single-sourced diagnostic tests and the balance between quality control and the urgency to fulfill the demand of an impending pandemic [5]. The diplomatic rows over medical supplies between European countries underlined the lack of international regulation on coordinating stockpiling rules, development of joint operational procedures, and essential item lists for collective responses [6]. At the same time, the lessons learned from the COVID-19 pandemic provide an important opportunity for global health development, warranting the need to outline the current gaps in the control of infectious diseases in global health.

**Fig. 1:**
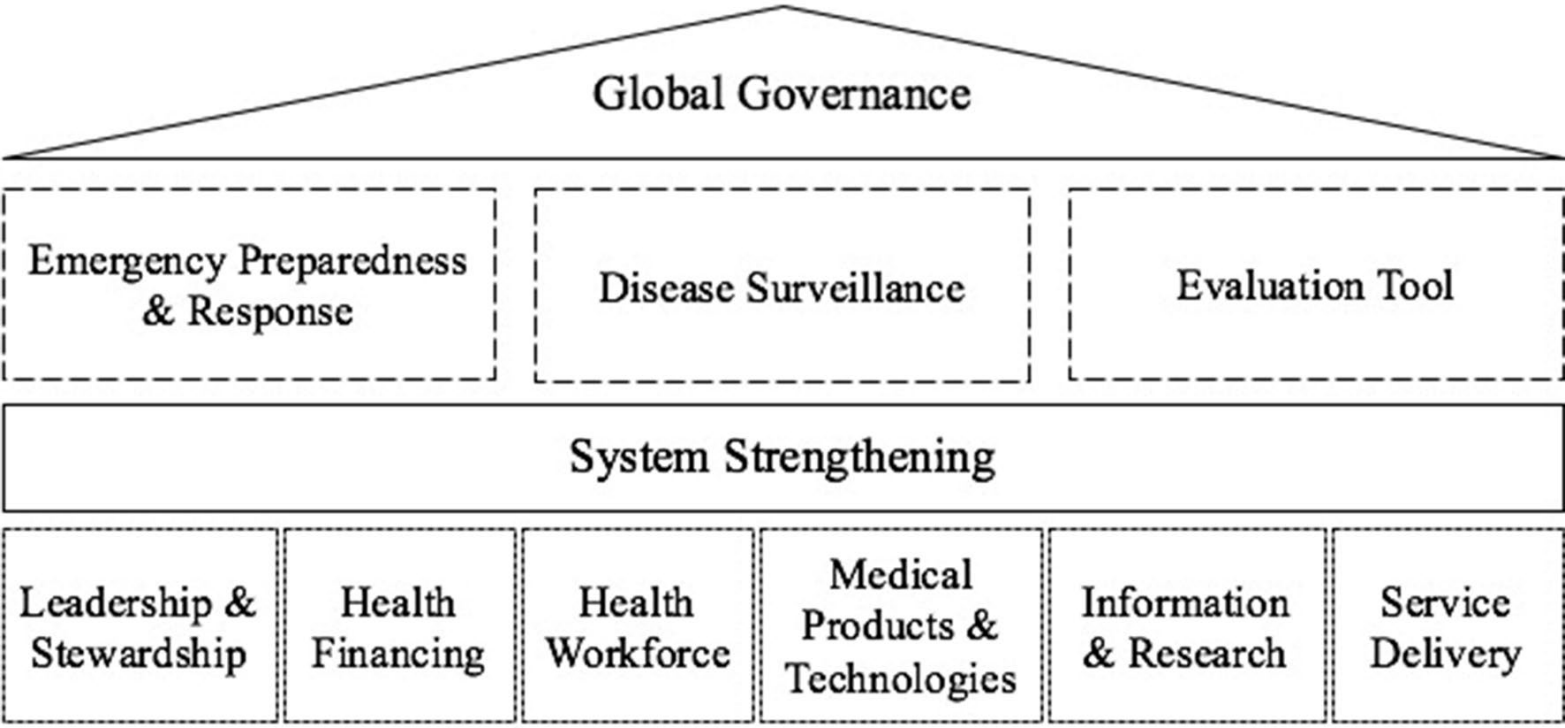
A framework for action against global infectious diseases

This paper outlines current deficiencies in infectious disease control under the purview of global health, including health systems, emerging preparedness and response, integrated surveillance, and evaluation tools (Fig. 1). Relevant literature was searched in multiple electronic databases (2010–2022), supplemented with manual searching (Additional file 1). Under the shadow of frequent pandemic outbreaks, we aim to raise awareness of global progress in infectious disease and to inform new developments in global health systems to address future threats worldwide.

## Current progress in infectious disease control

### Global governance

Ilona Kickbusch has proposed to analyze this space along three dimensions, namely “global health governance” (refers to “those institutions and processes of governance which are related to an explicit health mandate”), “global governance for health” (refers to “those institutions and processes of global governance which have a direct and indirect health impact”) and “governance for global health” (refers to “the institutions and mechanisms established at the national and regional level to contribute to global health governance and/or to governance for global health”) [7]. Many players have been involved, centered on the WHO and the World Bank because they represent the main sources of health expertise and development financing, respectively. In addition, a number of United Nations (UN) specialized agencies, funds, and programmes within the UN system, such as Food and Agriculture Organization (FAO), World Meteorological Organization (WMO), United Nations Environment Programme (UNEP), United Nations Population Fund (UNFPA), United Nations Development Programme (UNDP), have been playing important and various roles in global health. Global health governance also includes a wide variety of actors within the private sector and civil society. Some of those actors (e.g., the Bill & Melinda Gates Foundation) have become highly prominent in recent years. The leading role of global governance is still the international mechanism established by various sovereign countries [8], while more flexible informal international mechanisms also play an increasingly important role in setting the global health governance agenda.

### Emergency preparedness and response

Factors related to the capabilities for emergency preparedness include the capacity of the public health agency or the government to mobilize human, physical, and financial resources, to identify, prepare, and deploy staff, and to implement response operations and communicate with the public [9]. The WHO and its member states have recognized the significance of having a central location for emergency preparedness and response, aligning with the International Health Regulations (IHR) (2005) and Global Health Security Agenda. The WHO Department of Global Capacities, Alert, and Responses established the Public Health Emergency Operation Center (PHEOC) network in 2012 to promote best practices and support PHEOC capacity building among member countries. In 2020, the WHO requested the activation of the United Nations Crisis Management Policy by the UN Secretary-General, the highest level of crisis alert and the first activation for a health-related event. This policy activation enables the WHO to chair the COVID-19 Crisis Management Team and to coordinate UN strategies, policy decisions, and plans. In 2021, the WHO set up a new hub in Berlin, WHO Hub for Pandemic and Epidemic Intelligence, to ensure better global coordination in response to potential epidemics in the future. Meanwhile, some national strategies have also been developed to strengthen the capacity for emergency preparedness and response. For instance, Japan’s Ministry of Health, Labor, and Welfare established the Infectious Disease Emergency Specialists training in 2015, involving various national institutes with a broad range of expertise in health emergencies. In China, the Ministry of Emergency Management was established in 2018, with the overarching responsibility of preparing for and responding to natural and man-made disasters.

### Disease surveillance

Surveillance of communicable diseases covers communicable diseases and the pathogens attributable to the diseases. It includes both passive and active surveillance, such as syndromes, events, and other relevant factors such as drug sales and school attendance [10]. Based on surveillance data, timely and appropriate feedback facilitates early warning before, or early, in the epidemic/outbreaks of communicable diseases to inform the risk of spatial-temporal incidence and subsequent countermeasures. In 1968, the WHO underlined the significance of communicable diseases surveillance at the 21st World Health Assembly. Accordingly, countries started to establish surveillance and early warning systems for communicable diseases. The National Notifiable Diseases Surveillance System has been established in the United States to monitor infectious diseases, bioterrorism, and some non-communicable diseases, with electronic laboratory reporting for laboratory findings [11]. In China, the National Notifiable Diseases Reporting System achieves real-time and online reports of notifiable infectious diseases, recording the demography, clinical diagnosis, and epidemiological data of the cases. Similarly, the European Centre for Disease Prevention and Control has established the European Surveillance System in the European Union / European Economic Area. Additionally, modern surveillance systems use early warning technology to identify unusual increases in the incidence rate of certain communicable diseases beyond the normal level. They often incorporate spatial and temporal statistical alerts, custom querying, user-defined alert notifications, geographical mapping, remote data capture, and event communications [12]. Furthermore, the WHO established the Global Public Health Intelligence Network, which utilizes the non-governmental media data for early warning.

### Evaluation tool

The development of evaluation tools has played an increasingly crucial role in generating scientific evidence to understand the global reality and identify gaps and priorities in infectious disease control (Additional file 2). In 2007, the WHO proposed six core “building blocks” in its health system framework after the release of the World Health Report 2000 [13], which attempted to evaluate the performance of health systems for countries/territories around the globe and improve understanding of what a good health system should be. The WHO has also developed the IHR Monitoring and Evaluation Framework [14] to monitor progress according to the requirements of the IHR. Under the framework, the Self-Assessment Annual Reporting tool [15] has been designed for mandatory reporting, which quantifies a country’s progress in developing the capabilities of the 13 areas required in the IHR. In 2020, the WHO further developed the COVID-19 Strategic Preparedness and Response Monitoring and Evaluation Framework [16] to provide guidance for the COVID-19 response. An abundance of technical guidance has also been in place for managing infectious diseases. In 2020, the WHO released the road map and sustainability framework for neglected tropical disease (NTD) [17] to further guide the shift from a disease-specific approach to an integrated approach that cuts across all 20 NTD groups. In addition, a number of tools have been developed for the measurement of infectious disease control, such as the Global Burden of Disease tool [18], which opens up an era of the composite evaluation of disease burden and serves as one of the most recognized tools for quantifying global health losses from diseases, injuries, and risk factors, and the Performance of Veterinary Services Pathway, which provides data resources for the analysis of gaps and capacities in zoonotic diseases control.

### System strengthening

The most fundamental approach to infectious disease prevention and control is to enhance health system resilience. A health system, as modularized by the WHO, consists of six core components or “building blocks” [19]. Firstly, leadership/governance and health information systems serve as the basis for the other blocks. Local and central governments’ leadership, intergovernmental coordination, infectious disease-related legislation, and information communication are identified as key factors for risk management against infectious diseases [20]. Secondly, flexible, accessible financing is important for maintaining a resilient health system that is prepared for infectious disease prevention and control. In 2019, according to the statistics from Global Health Expenditure Database [21], the Domestic General Government Health Expenditure (GGHE-D) reached a global average of USD 26.9 billion, and the expenditure on infectious and parasite diseases took up 35.12% of the total GGHE-D. Health system strengthening and sector-wide approaches draw an overall increasing investment worldwide and in 2020 reached USD 5.5 billion, or 9.95% of the total spending [22]. Thirdly, the role of health workforce for a competent health system has been emphasized as central during the COVID-19 pandemic [23]. Finally, access to health products, technology, and service delivery, as the immediate outputs of a health system [24], constitute people’s direct impression and experience of the health system, calling for strengthening of human-centric healthcare delivery. Along the road, primary health care (PHC) is deemed “the engine for Universal Health Coverage (UHC)” [25] and essential to ensure a resilient health system because it is effective in reducing infectious disease mortality and improving health outcomes [26].

## Advances in key infectious diseases

Due to significant efforts made in enhancing the health system, surveillance capacity, and disease management, the 21st century has been marked by a substantial decline in the global disease burden of infectious diseases, which have been elaborated through the cases of influenza, malaria, tuberculosis and NTDs in this section.

### Surveillance system building for influenza control

The WHO Global Influenza Surveillance Network was founded in 1952. It was renamed the Global Influenza Surveillance and Response System (GISRS) in 2011 with the establishment of the Pandemic Influenza Preparedness Framework designed for the sharing of influenza viruses, access to influenza vaccines, and related benefits. In the past decades, the surveillance system has grown to comprise 143 National Influenza Centers, 6 WHO Collaborating Centers, 4 Essential Regulatory Laboratories, and 13 H5 Reference Laboratories [27]. During the COVID-19 pandemic, the GISRS contributed to the surveillance of COVID-19, including laboratory testing, genomic sequencing, data sharing with the Global Initiative on Sharing All Influenza Data, and surveillance data generation for global platforms [28]. In 2021, the WHO released the Global Genomic Surveillance Strategy for Pathogens with Pandemic and Epidemic Potential 2022–2032, in order to strengthen genomic surveillance and scale for quality, timely, and appropriate public health actions within local to global surveillance systems. The majority of respiratory infections are mild or asymptomatic, which may be neglected by existing surveillance systems. Because of this, a fast and flexible surveillance system has been developed called Influenzanet [29]. Influenzanet collects baseline information on volunteers and conducts follow-ups during flu seasons, including flu-related symptoms and medical visits. Moreover, Influenzanet has the advantage of collecting personal data, which can be used to identify risk factors associated with influenza incidence. The data architecture of the system also allows for extended data collection to monitor other common or emerging communicable diseases, in addition to influenza.

### Health service delivery for malaria control

Fever is a common symptom of malaria in children, and the number of “children under age 5 with fever for whom advice or treatment was sought” within 2 weeks before the survey is an important indicator set as a goal in the *Sierra Leone Malaria Control Strategic Plan* [30]. In 2010, to remove the cost barrier of maternal and child health services and increase the effect of UHC and PHC, the government of Sierra Leone implemented the Free Health Care Initiative (FHCI), covering essential care for pregnant women, lactating women and children under age 5 [31]. With the widely-distributed Peripheral Health Units including maternal and child health posts covering 500–5000 population, community health posts covering 5000–10,000 population, and community health centers covering 10,000–30,000 population, and the growing community health workforce boosted by the National Health Worker Program that was established in 2012 and supported by donors such as Global Fund and World Bank [32], the care-seeking percentage among children under 5 with fever after the implementation of FHCI was reported to be at least 1.4 time higher than before the FHCI [31].

### Medical product provision for tuberculosis control

Since 2005, when the partnership between the Global Fund and Sudan was established, a considerable proportion of funding for tuberculosis has been allocated to health product provision and distribution [33]. Among the numerous programs is the procurement and installation of GeneXpert machines. GeneXpert is a molecular diagnostic testing technology for multidrug-resistant tuberculosis that can reduce laboratory processing time to less than one day [34], which is much more condensed than the processing time of traditional testing methods that can take weeks. In 2015, the installation of GeneXpert machines, together with comprehensive training for lab technicians and rehabilitation of tuberculosis laboratories, started first in Khartoum, the Capital of Sudan, then in all the other states. In 2019, there were 72 GeneXpert machines in the country, 52 of which were functioning [35]. Accompanied by other investments and programs in health products, human resources, technical assistance, etc., the efforts of donors from all over the world and the government of Sudan has led to an increase in tuberculosis diagnoses and a decrease in mortality.

### Integrated management for NTDs control

NTDs affect approximately one billion of the world’s poorest people and should not be neglected. Since the WHO released the first neglected tropical disease road map in 2010, the World Bank, major pharmaceutical companies, bilateral aid agencies, endemic countries, and other public and private sector organizations increased their support for the global neglected tropical disease response. The new roadmap for neglected tropical diseases 2021–2030 proposes concrete actions focused on integrated platforms for the delivery of interventions and will thereby improve program cost-effectiveness and coverage. Therefore, integrating NTD control efforts at the national level will improve the accountability, efficiency, and cost-effectiveness of programs as many of them have similarities in their epidemiology and control measures [36]. In this case, national and local governments must lead work to define their elimination agendas and realize objectives clearly, financed partially or fully through domestic funds as they are both the drivers and the beneficiaries of the NTDs’ elimination.

## Open challenges in global infectious disease control

Despite the progress achieved, the discordance among government actors and absent data sharing platforms or tools has led to significant challenges ahead in global infectious disease control.

Firstly, challenges remain in constructing a well-structured global health governance mechanism. With the diversification of global health determinants and the increasing number and influence of actors in global health governance [37], the coordination of global health governance tends to be fragmented, leading to poor global health governance. Additionally, politicizing health issues to pursue a country’s political goals is inconsistent with the goal of global health governance and global health cooperation. A pilot of a “One United Nations” reform has had positive results but still doesn’t address a plethora of non-UN actors who organize projects and send delegations [38].

Secondly, the COVID-19 pandemic exposed deficiencies in emergency preparedness and response systems for each country and the regional and global entities as a whole. While the value of emergency preparedness and response has already been demonstrated [39], some policymakers balk at investment in preparedness efforts once the immediate threat subsides [40]. Insufficient resilience and fragility of health systems diminishes the effectiveness of infectious disease prevention and control measures [41], so discordance in preparedness policy has led to unfulfilled targets in health system resilience and a capacity gap in infectious disease response capacity [42].

Thirdly, challenges remain in data sharing and the design of a global disease surveillance system. In the Global Outbreak Alert and Response Network (GOARN) [43] framework, a lack of bilateral and multilateral cooperation hindered progress and transparency, failing to ensure timely and complete information sharing among partners. The experiences in response to the H7N9 avian flu and COVID-19 highlighted the difficulty in the cross-sectoral corporation between health administration and agricultural sectors, and increasing evidence found that changes in climate and land use will facilitate zoonotic spillover from wild mammals to humans [44]. Furthermore, it is necessary to increase the timely and full sharing of sequence data and support for countries with limited sequencing and bioinformatics capacity.

Fourthly, there remain challenges in developing tools for global health evaluation and monitoring. The COVID-19 outbreak has stressed the importance of addressing global health threats from a system-wide perspective; thus, systematic evaluation frameworks are needed to integrate fragmented evidence for policymakers to determine priorities in the larger picture of decision-making practice. Furthermore, the data quality for global evaluation is inadequate, and inconsistency may occur between different sources as many of the existing data are self-reported. Technological advances are needed to improve the data quality and methodology of data integration and comprehensive analysis, especially for multi-dimensional data with diverse formats and scales [45].

## Looking forward

In recognizing the challenges, urgent actions are needed with deep multi- and cross-sectoral cooperation to break down barriers under the purview of global health.

First and foremost, global health warrants a need for sustainable capacity-building efforts in emergency preparedness and response. The investments in the infrastructure and human capital for emergency preparedness and response must be continuous. Such investments, if delayed until emergencies occur, will be much less effective. Moreover, clear and effective communication strategies need to be implemented, balancing the triple objectives of keeping the public informed, minimizing panic and circulation of misinformation, and promoting ethical and effective public health policies and interventions. In this process, countries should examine their legal framework to prepare for emergencies; WHO should promote knowledge exchange in this area and also take the responsibility of strengthening IHRs related to emergency preparedness and response.

Furthermore, a systematic redesign should be considered to enhance the resilience of health systems. To strengthen infectious disease prevention and control, PHC coverage and health product and technology accessibility require immediate promotion; in the meantime, all upper-stream “building blocks” of the health system should be taken into consideration. For low- and middle-income countries, financing is a major weakness, and this is where donor countries and international organizations come into play. The quantity and quality of the workforce developed based on policy, investment, and domestic/imported experience are also crucial for ensuring the output and impact of PHC, while health surveillance and survey systems allow policy-makers to grasp situations and make timely adjustments.

Meanwhile, it is important to combine the surveillance of zoonotic pathogens, animal diseases, and local biodiversity, using developments in data integration in the concept of One Health that addresses shared health threats at the human-animal-environment interface. One example is hepatitis E virus (HEV), which is a notifiable infectious disease in China [46]. Several animal HEV genotypes emerged to infect humans, such as HEV-7 and HEV-C1, along with an expanding range of animal hosts for HEV. Therefore, a comprehensive surveillance system was initiated in 2001 [47]. Currently, surveillance has developed into a multi-dimensional monitoring framework, which characterizes a framework of health/at-risk/occupationally-exposed/infected populations, environmental determinants, and risk factors including animals and animal products.

Lately, the research team from Shanghai Jiao Tong University developed a global One Health index (GOHI) [48, 49], which is used to identify the current gaps in One Health practice for countries and territories and guide the formulation of effective measures in local settings. It has been indicated that there is an imminent need to establish a comprehensive database that incorporates multiple components, including people, animals, and the environment. While improving the quantity and quality of data, it is important to establish a data-sharing mechanism. Monitoring information from multiple sources including traditional systems, dedicated systems for certain diseases, populations, animals and plants, and environmental data, would be combined and integrated to formalize a comprehensive system. In addition, the system should coordinate the surveillance and early warning systems across regions to promote effective implementation. An intelligent multi-point trigger mechanism, including symptoms, events, media, academic publications, and determinants (high-risk behavior and vectors), should be implemented, which warrants appropriate methods for data integration and a complex algorithm for analysis.

## Conclusions

Lessons learned from COVID-19 have shown that, without collaboration and coordination, pandemics may prevail by taking advantage of the weakest link in our connected world. The evidence shared in this article serves as a foundation for gap identification and policy improvement in global infectious diseases, aiding preparations for the next pandemic. Moreover, monitoring and surveillance should be considered in human/animal, in a joint database, whereby action in human can be based on a threshold set in animal. It has been highlighted that countries and international organizations must overcome geopolitical differences to coordinate responses to prepare for future emergencies. Ways to achieve such developments in the context of limited health resources and differentiated political, social, and economic backgrounds remain a problem, which demands enhanced cross-sectoral and multi-disciplinary efforts in promoting effective institutional communication and enabling collaboration and capacity building among actors in the arena.

### Supplementary Information

**Additional file 1**: Representative works in global health governance.

** Additional file 2**: Selected evaluation frameworks and tools for global health research and practice.

## Data Availability

Not applicable.

## Abbreviations

COVID-19: Coronavirus disease 2019
GGHE-D: Domestic General Government Health Expenditure
FAO: Food and Agriculture Organization
FHCI: Free Health Care Initiative
GISRS: Global Influenza Surveillance and Response System
GOHI: Global One Health index
GOARN: Global Outbreak Alert and Response Network
HEV: Hepatitis E virus
IHR: International Health Regulations
NTD: Neglected tropical disease
PHC: Primary health care
PHEOC: Public Health Emergency Operation Center
UN: United Nations
UNDP: United Nations Development Programme
UNEP: United Nations Environment Programme
UNFPA: United Nations Population Fund
UHC: Universal Health Coverage
WHO: World Health Organization
WMO: World Meteorological Organization
