# Supplementary material for: Infectious disease control: from health security strengthening to health systems improvement at global level
Source: Glob Health Res Policy. 2023 Sep 5;8:38. doi: 10.1186/s41256-023-00319-w (PMC10478312; doi:10.1186/s41256-023-00319-w)
Supplement: Supplementary file 1 — Additional file 1: Representative works in global health governance. [file 41256_2023_319_MOESM1_ESM.docx]

**Additional file 1**

**Appendix 1 –Selected Articles**

Table A1 Representative works in global health governance

| Title | Year | Journal | Design | Topic | Contents | Conclusion |
| --- | --- | --- | --- | --- | --- | --- |
| [Global health diplomacy-reconstructing power and governance](https://pubmed.ncbi.nlm.nih.gov/35594877/) | 2022 | Lancet | systematic review | global health diplomacy | Concepts and theories History  The present phase | In a changing world, new international relations concepts and theories are called for to inform global health diplomacy. |
| [The Novel Coronavirus Originating in Wuhan, China: Challenges for Global Health Governance](https://pubmed.ncbi.nlm.nih.gov/31999307/) | 2020 | JAMA | review | global health governance under covid-19 | Current Status Control Measures in China Control Measures by Governments Worldwide Nonpharmaceutical Interventions Role of the WHO Bringing 2019-nCoV Under Control | Managing the outbreak requires international cooperation using traditional public health strategies, coordinated international response, and act decisively. |
| [Hindsight is 2020? Lessons in global health governance one year into the pandemic](https://pubmed.ncbi.nlm.nih.gov/33723454/) | 2021 | Nature Medicine | review | global health governance under covid-19 | Science facts about COVID-19;  Role of the WHO; National governance (overall strategic difference; public health measures; social inequalities; leadership and communication; economy versus health) | Collective progress will be dependent on a coordinated global effort to leave no one behind. |
| [Reimagining Global Health Governance in the Age of COVID-19](https://pubmed.ncbi.nlm.nih.gov/33026872/) | 2020 | American Journal of Public Health | editorial | global health governance under covid-19 | Bring nations together; Obstacles to global solidarity; governance to realize solidarity (A Diminished United States; A Fractured Global Health Ecosystem; A Changing World Health Organization) | Global health governance is at a crossroads, necessitating a new governance model that works cooperatively through shared governance. |
| [Public health emergency preparedness: a framework to promote resilience](https://pubmed.ncbi.nlm.nih.gov/30518348/) | 2018 | BMC Public Health | qualitative | emergency preparedness | **Methods:** structured Interview Matrix facilitation technique;  **Participants:** six focus groups across Canada (practitioners from public health and related sectors);  **Objective:** to describe the essential elements of a resilient public health system and how the elements interact as a complex adaptive system | **Twelve essential elements of public health emergency preparedness**: Governance and leadership; Planning process; Collaborative networks; Community engagement; Risk analysis; Surveillance and monitoring; Practice and experience; Resources; Workforce capacity; Communication; Learning and evaluation |
| [Evaluating integrated surveillance of antimicrobial resistance: experiences from use of three evaluation tools](https://pubmed.ncbi.nlm.nih.gov/32213319/) | 2020 | Clinical Microbiology and Infection | review | Integrated antimicrobial resistance (AMR) surveillance | Strengths and weaknesses of 3 evaluation tool;  Guidance on how to choose a fit-for-purpose tool. | All three tools address multiple decision-making levels and aspects of stakeholder engagement. |
| [Conceptual analysis of health systems resilience: A scoping review](https://pubmed.ncbi.nlm.nih.gov/31100697/) | 2019 | Social Science & Medicine | review | health systems resilience | **Method:** a scoping review **Objective:** to describe the state of knowledge in this area | The current conceptualization of health systems resilience is too scattered to enable the enhancement of this concept with great potential, opening a large avenue for future research. |
| [Defining and classifying public health systems: a critical interpretive synthesis](https://health-policy-systems.biomedcentral.com/articles/10.1186/s12961-020-00583-z) | 2020 | Health Research Policy and Systems | review | public health systems | **Objective:** to define public health systems and assess differences between healthcare systems and public health systems within established health systems frameworks. **Methods:** A critical interpretive synthesis. Data extraction, coding and analysis followed a best-fit framework analysis method. | Public health systems are unique and vital entities within health systems. Establishing the scope of public health is crucial to understanding its role within the larger health system and adds to the discourse around the relationship between public health, healthcare and population health. |
| [Evaluation of global health capacity building initiatives in low-and middle-income countries: A systematic review](https://pubmed.ncbi.nlm.nih.gov/33110574/) | 2020 | Journal of Global Health | systematic review | evaluation of Global Health Capacity Building (GHCB) | **Methods:** This study systematically reviews evaluation approaches for GHCB initiatives in LMICs by searching 4 databases for studies reporting evaluation of a GHCB initiative in a LMIC from January 1, 2009 until August 15, 2019. **Results:** 63 articles were eligible for analysis. Most studies stemmed from Africa and Asia (69.8%), were delivered and evaluated face-to-face (74.6% and 76.2%), mainly to professionals (57.1%) and community workers (20.6%). | Standardization of evaluations is called for, especially for long-term and wider impact assessment of online and blended modalities. |
| Emerging infectious disease prevention: Where should we invest our resources and efforts? | 2019 | Journal of Infection and Public Health | review | emerging infectious disease prevention | Targets for investments in emerging infectious disease prevention  Pivotal considerations and perspectives: Strengthen the basics and invest in new technologies | Human and monetary investments focused on  animals, humans, and vectors meet different demands and support the elucidation of different questions in the emerging infectious diseases context. |
