# Supplementary material for: Infectious disease control: from health security strengthening to health systems improvement at global level
Source: Glob Health Res Policy. 2023 Sep 5;8:38. doi: 10.1186/s41256-023-00319-w (PMC10478312; doi:10.1186/s41256-023-00319-w)
Supplement: Supplementary file 2 — Additional file 2: Selected evaluation frameworks and tools for global health research and practice. [file 41256_2023_319_MOESM2_ESM.docx]

**Additional file 2**

**Appendix 2 – Evaluation tools for global health**

Table A2 Selected evaluation frameworks and tools for global health research and practice

| Title | Category | Scope | Components | Developed by | Year |
| --- | --- | --- | --- | --- | --- |
| Six Building Blocks of Health System | Conceptual Framework | Structure of Health System (Human) | service delivery, governance and stewardship, health system financing, human resources, medical products and technology, health information system | World Health Organization | 2007 |
| Control Knob Framework | Conceptual Framework | Structure of Health (Financing) System (Human) | control knobs (financing, payment, organization, regulation, behavior), performance outcomes (health status, consumer satisfaction, risk protection) | World Bank | 2014 |
| Monitoring and Evaluation Framework | Reporting Framework | IHR Implementation and COVID-19 Control | 3 geographical scopes (global level, countries and priority countries), 3 planning and monitoring needs (preparedness, response and situation), 9 pillars/areas (covering country-level action, travel management, national laboratories, infection prevention and control, case management, logistics, essential health services and systems, cross-cutting issues) | World Health Organization | 2020 |
| Sustainability Framework for Action against NTDs | Action Framework | NTD Control | module of contextual inputs (political environment, epidemiology and social context), module of intervention (non-health sector, health system), module of outcome (coverage of service, roadmap outcome realization) | World Health Organization | 2021 |
| Global Technical Strategy Framework for Malaria 2016-2030 | Action Framework | Malaria Control | 3 pillars (ensure the universal access to malaria care service, accelerate the elimination, and transform malaria surveillance into core intervention), 2 supporting elements (research and enabling environment) | World Health Organization | 2017 |
| Global Burden of Disease | Evaluation Tool | Disease Burden | YLL (Years of Life Lost), YLD (Years of Life Lived with Disability) | GBD Research Team | Annual |
| States Parties Annual Reporting | Evaluation Tool | IHR Implementation | 13 key IHR areas (IHR legislation and financing, IHR strategic coordination, zoonotic events and the human-animal interface, food safety, laboratory, surveillance, human resources, emergency preparedness for response, health service provision, risk communication, points of entry, chemical events, radiation emergencies) | World Health Organization | Annual |
| Performance of Veterinary Services | Evaluation Tool | Veterinary Service | 4 fundamental components(human physical and financial resources, technical authority and capacity, interaction with stakeholders, access to markets) and 45 critical competencies | World Organization for Animal Health | 2007- |
| Global One Health Index | Evaluation Tool | One Health | external driver index (earth system, economical system, technological system, sociological system and institutional system), intrinsic driver index (human health, animal health and environment health), core driver index (zoonotic diseases, food security, antimicrobial resistance, climate change and governance) | GOHI Research Team | 2022- |
